# Refractory Mucocutaneous Infections by Herpes Simplex Virus (HSV) in Hematopoietic Cell Transplant Recipients: A Real-world, Multicenter Study

**DOI:** 10.1093/ofid/ofag377

**Published:** 2026-06-19

**Authors:** Genovefa A Papanicolaou, Yeon Joo Lee, Tali Shafat, Yuxuan Li, Jo-Anne H Young, Amy Spallone, Karam M Obeid, Gunjan L Shah, Patrick W Flaherty, Mandeep K Sekhon, Kitty Chiu, Veronica Dioverti, Sarah P Hammond, Sanjeet Dadwal, Joshua A Hill, Camille N Kotton, Veronica Miller, Per Ljungman, Roy F Chemaly

**Affiliations:** Infectious Diseases and Allergy Service, Department of Medicine, Memorial Sloan Kettering Cancer Center, NewYork, New York, USA; Department of Medicine, Weill Cornell Medicine, Cornell University, NewYork, New York, USA; Infectious Diseases and Allergy Service, Department of Medicine, Memorial Sloan Kettering Cancer Center, NewYork, New York, USA; Department of Medicine, Weill Cornell Medicine, Cornell University, NewYork, New York, USA; Department of Infectious Diseases, Infection Control and Employee Health, The University of Texas MD Anderson Cancer Center, Houston, Texas, USA; Infectious Diseases and Allergy Service, Department of Medicine, Memorial Sloan Kettering Cancer Center, NewYork, New York, USA; Division of Infectious Diseases and International Medicine, University of Minnesota, Minneapolis, Minnesota, USA; Department of Infectious Diseases, Infection Control and Employee Health, The University of Texas MD Anderson Cancer Center, Houston, Texas, USA; Division of Infectious Diseases and International Medicine, University of Minnesota, Minneapolis, Minnesota, USA; Department of Medicine, Weill Cornell Medicine, Cornell University, NewYork, New York, USA; Adult Bone Marrow Transplantation Service, Department of Medicine, Memorial Sloan Kettering Cancer Center, NewYork, New York, USA; Vaccine and Infectious Disease Division, Fred Hutchinson Cancer Center, Seattle, Washington, USA; Vaccine and Infectious Disease Division, Fred Hutchinson Cancer Center, Seattle, Washington, USA; Division of Infectious Diseases, City of Hope, Duarte, California, USA; Division of Infectious Diseases, Johns Hopkins University, Baltimore, Maryland, USA; Division of Infectious Diseases, Massachusetts General Hospital, Harvard Medical School, Boston, Massachusetts, USA; Mass General Brigham Cancer Institute at Massachusetts General Hospital, Boston, Massachusetts, USA; Division of Infectious Diseases, City of Hope, Duarte, California, USA; Vaccine and Infectious Disease Division, Fred Hutchinson Cancer Center, Seattle, Washington, USA; Division of Infectious Diseases, Massachusetts General Hospital, Harvard Medical School, Boston, Massachusetts, USA; Forum for HIV Collaborative Research, University of California, Berkeley, California, USA; Department of Cellular Therapy and Allogeneic Stem Cell Transplantation and Department of Infectious Diseases, Karolinska University Hospital Huddinge, Karolinska Comprehensive Cancer Center, Stockholm, Sweden; Department of Medicine Huddinge, Division of Hematology, Karolinska Institute, Stockholm, Sweden; Department of Infectious Diseases, Infection Control and Employee Health, The University of Texas MD Anderson Cancer Center, Houston, Texas, USA

**Keywords:** acyclovir resistance, foscarnet, hematopoietic cell transplant, herpes simplex virus (HSV), refractory infection

## Abstract

**Background:**

We examined treatment patterns and clinical outcomes of adult hematopoietic cell transplant (HCT) recipients with refractory mucocutaneous herpes simplex virus (HSV) infection from 7 US centers.

**Methods:**

Multicenter, retrospective study of laboratory confirmed HSV infection and failure to improve after ≥7 days of appropriately dosed anti-HSV therapies. Resistance testing was performed at clinicians' discretion. Therapies were captured for the first refractory HSV infection post-HCT (index episode). Time to complete lesion healing, toxicities, and HSV recurrence up to 1 year from index HSV episode lesion healing were extracted from electronic medical records. The cumulative incidence function was used to estimate time to healing with death as competing risk.

**Results:**

Of 125 patients, acyclovir resistance was confirmed in 85/104 patients (81.7%) tested. First therapy was nucleoside analog in 100 patients and foscarnet in 25. A total of 110 patients (88%) received second line therapy(ies), including foscarnet (94), intravenous cidofovir (6), and/or topical antivirals (21). Mean treatment durations (SD) were 21.8 days (13.8), 20.3 (10.1), and 41.0 (37.5) for foscarnet, cidofovir, and topicals, respectively. Complete healing occurred in 69 patients (55.2%) at a median of 38 days (range 257) from the anti-HSV therapy start. Nephrotoxicity was reported during 40.4% of foscarnet and 50.0% of cidofovir courses, respectively. Among 69 patients with completed healing, 27 (39.1%) experienced HSV recurrence during follow up.

**Conclusions:**

Our study highlights the challenges of managing refractory HSV post-HCT during the study timeframe, and underscores the unmet need for safer, more effective, and orally bioavailable therapies for refractory HSV.

## Teaser

A total of 55.2% HCT recipients with refractory HSV healed completely at a median of 38 days of therapy. Seventy-five percent of patients received foscarnet. Renal function abnormalities were reported in 40.4% of foscarnet courses. Effective and better-tolerated therapies are needed for refractory HSV.

Mucocutaneous infections resulting from herpes simplex virus (HSV) in hematopoietic cell transplant (HCT) recipients tend to be more aggressive, extensive, and persistent compared to immunocompetent individuals due to impaired immunity, cytopenia, poor nutritional status, graft-versus-host disease, and immunosuppressants [1, 2]. Severe oral HSV infections may impede adequate oral intake and require hospitalization for hydration, analgesics, and intravenous (IV) antiviral therapy, adversely impacting patients' quality of life and increasing health care resource utilization. In addition, HSV infections developing on acyclovir prophylaxis may be resistant to acyclovir [3–6]. Antiviral resistance by phenotypic assays take weeks to perform; thus, results are not available in real time for treatment decisions [7].

According to recently developed definitions for clinical trials, refractory HSV is defined as no clinical improvement of HSV lesion(s) or new lesions appearing after at least 7 days of standard dose nucleoside analog (NUC) treatment (excluding prophylaxis and suppressive therapy). Resistant HSV is defined as refractory plus laboratory-confirmed antiviral resistance [8]. Acyclovir resistance occurs in up to 14% of HCT recipients, with estimates varying widely across studies [8]. Real-world studies on the burden of refractory or resistant HSV in HCT or people with HIV remain limited [2, 9–11]. Larger studies are needed to capture differences in patient characteristics and contemporary clinical practices. Non-NUCs (second-line therapies) include mainly foscarnet, IV cidofovir, or topical cidofovir and topical imiquimod. Foscarnet or IV cidofovir are usually active against acyclovir-resistant HSV [5, 9] but have treatment-limiting toxicities, mainly nephrotoxicity and requirement for intravenous administration [2, 9, 11–14]. Topical antiviral preparations may be occasionally used as an adjunct to systemic antivirals or as step-down therapy, with scant supporting evidence [10, 12, 15, 16]. Clinical responses with continuous infusion of high-dose IV acyclovir have also been described in a case series [17, 18].

Effective, safer, and orally bioavailable therapies are critically needed to improve the quality of life of HCT patients with refractory HSV infections [12, 19, 20]. Small molecules targeting the helicase primase complex currently in clinical development are orally bioavailable and retain antiviral activity against acyclovir-resistant HSV-1 and HSV-2 strains [21]. Amenamevir is approved in Japan for treatment of herpes zoster and episodic treatment of recurrent HSV [22, 23]. Recently, pritelivir demonstrated superior efficacy and favorable tolerability compared with standard of care therapies in immunocompromised patients with refractory HSV infection in a global phase 3 study (Clinicaltrials.gov: NCT03073967) [24].

This real-world study aims to (1) describe antiviral treatment patterns and (2) report clinical outcome of refractory HSV infections among HCT recipients with management strategies available during the study timeframe.

## METHODS

### Study Population

This multicenter, retrospective study was conducted in 7 academic transplant centers in the United States. The study was approved by the institutional review board at each participating center. Deidentified patient-level data were entered into a computerized database and analyzed by the coordinating center (Memorial Sloan Kettering Cancer Center). Refractory HSV cases were identified at each institution through review of institutional databases and electronic medical records for patients transplanted from 2009 through 2024. Adult (≥18 years) HCT with laboratory-confirmed mucocutaneous HSV infection (by polymerase chain reaction, viral culture, or immunohistochemistry) were eligible if they did not improve after ≥7 days of appropriately dosed anti-HSV therapy and received second line therapies or high-dose IV acyclovir unless at physicians' or patients' discretion they were not deemed candidates for second line therapy. Patients who participated in the phase 3 trial of pritelivir (Clinicaltrials.gov: NCT03073967) were excluded. Patients were followed up to 12 months from healing of the index HSV episode or until death or censoring, whichever occurred first.

### Definitions

The index HSV episode is the first refractory HSV episode after HCT. Treatment index date was the start date of anti-HSV therapy for the index episode. Baseline characteristics were assessed at the time of index HSV diagnosis. NUC therapies include standard treatment doses of acyclovir, valacyclovir, famciclovir, valganciclovir, and high-dose IV acyclovir.

Second-line therapies (non-NUC) include foscarnet, IV cidofovir, topical cidofovir, topical imiquimod, leflunomide, or brincidofovir (Supplementary Table 1).

Resistant HSV is defined as refractory HSV infection plus laboratory-confirmed resistance by phenotypic or genotypic assays to acyclovir and/or foscarnet during the index HSV episode. The inhibitory concentration (IC_50_) cutoffs for acyclovir and foscarnet were determined according to each institution's laboratory interpretive criteria.

Date of healing was defined as the first date when complete healing of all lesions was documented in standard of care assessments at each center.

Recurrent HSV was defined as appearance of new, laboratory-confirmed HSV lesions >7 days after complete healing of the index episode until death or last follow up, up to 1 year from healing of the index refractory episode.

#### HSV Therapy

Any antiviral with anti-HSV activity that was administered prior and up to the HSV diagnosis is considered prophylaxis regardless of dose and indication. For example, ganciclovir or valganciclovir administered for cytomegalovirus prior to HSV diagnosis was considered HSV prophylaxis regardless of dosing regimen.

A treatment course for HSV was defined as administration of anti-HSV therapy for >2 consecutive days. The first antiviral course started at the treatment index date. Sequential courses are labeled numerically starting from the treatment index date. Centers entered the dose and frequency for each antiviral based on the intended dose for normal renal function. Addition or removal of antiviral(s) to ongoing therapy was considered a distinct course.

Toxicity and intolerance events emerging during each antiviral course were extracted from routine clinical assessments in the electronic medical records. Broad categories of toxicities were listed in the case report form, such as renal function abnormalities, electrolyte abnormalities, myelosuppression, neutropenia, thrombocytopenia, and nausea. Other events were reported as free text. Discontinuation because of toxicity at the end of each antiviral course was also extracted from the progress notes.

#### Objectives and Endpoints

Our primary endpoint was complete healing of all lesions from the treatment index date.

Our first objective was to describe antiviral treatment patterns for the index HSV episode including types and sequencing of antivirals and total treatment days for each antiviral until complete healing, death, or censoring.

Our secondary objectives were to: (1) estimate the time to complete lesion healing; (2) describe toxicities associated with foscarnet or cidofovir; and (3) estimate the incidence of HSV recurrence up to 1 year from healing of the index HSV episode among patients who achieved complete healing.

### Statistical Analyses

Descriptive statistics were used to summarize data. HSV antiviral treatments from the treatment index date to the initiation of foscarnet and/or cidofovir were summarized and visualized using a Sankey diagram to show the transitions across antiviral therapies. Each Sankey node represents HSV treatment groups, and each link represents the number of patients transitioning from 1 treatment category to the next. Link weights were computed as patient counts and displayed as flow widths. Counts were embedded in the node labels. Node appearance was formatted to maintain consistent color mapping across treatment categories.

Total treatment days for each antiviral are summarized. The mean duration of each antiviral is calculated as total antiviral days divided by the number of patients receiving the given antiviral agent. Continuous variables were summarized using median and first and third quartiles (Q1, Q3). Categorical variables were summarized using absolute and relative frequencies.

The cumulative incidence function was applied to estimate time to complete healing of the index HSV episode. Patients that discontinued anti-HSV treatment before healing or being lost to follow up were censored at the time of last anti-HSV therapy (excluding pritelivir). Patients who participated in the phase 2 clinical trial of pritelivir (Clinicaltrials.gov: NCT03073967) or received pritelivir under an expanded access program were censored on the day of starting pritelivir. Death was considered a competing risk.

Kernel density estimation was used to visualize the distribution of time to complete lesion healing. The density curve represents smoothed estimate of the underlying probability distributions. The total area under the density curve equals 1, and the area under the curve over a specified time interval corresponds to the proportion of patients with healing times within that interval. Higher density indicates greater clustering of healing times around a given day.

All analyses were conducted using R, version 4.2.1 (R Foundation for Statistical Computing, Vienna, Austria). The Sankey diagram was generated in Python (v3.13.9) using Plotly (v6.3.0; plotly.graph_objects).

## RESULTS

### Study Population

One hundred and twenty-five patients were analyzed. Ninety patients (72%) received HCT from January 2017 through December 2024 (contemporary cohort). Sixteen patients were censored before compete lesion healing, including 4 patients treated with pritelivir and 12 patients who discontinued anti-HSV treatment or were lost to follow up before healing.

### Baseline Characteristics

The index HSV episode was diagnosed at a median of 101 days from HCT (Q1, Q3: 28, 351; minimum: −8, maximum 4116). Table 1 summarizes the demographics and clinical characteristics at diagnosis of the index HSV episode. Thirty percent of patients received T-cell depleted HCT (CD34^+^ selection, antithymocyte globulin, or alemtuzumab) and 32.8% received posttransplant cyclophosphamide. At HSV diagnosis, 24.0% patients were on prednisone ≥20 mg/day or equivalent, 35.2% had relapse or progression of their underlying malignancy, and 27.2% were neutropenic. All patients were on HSV prophylaxis at the onset of the index episode (Table 1).

**Table 1.** Baseline Characteristics of the 125 Patients

Characteristic	…	N (%)
Median age (minimum—maximum), years	52 (20–82)	
Gender	Male	71 (56.8)
Indication for HCT	Leukemia	70 (56.0)
	Myelodysplastic syndrome	13 (10.4)
	Lymphoma	22 (17.6)
	Myeloma	10 (8.0)
	Other^a^	10 (8.0)
Donor type	Matched related or unrelated	56 (44.8)
	Haploidentical	31 (24.8)
	Mismatched related or unrelated	32 (25.6)
	Autologous	6 (4.8)
T-cell depletion	…	…
	PTCy ± ATG^b^	41 (32.8)
	Ex-vivo T-cell depletion ± ATG^c^	18 (14.4)
	ATG	14 (11.2)
	Alemtuzumab ± other^d^	5 (4.0)
	Unknown	2 (1.6)
Relapse/progression of underlying malignancy	…	44 (35.2)
Prednisone or equivalent ≥20 mg/day	…	30 (24.0)
Baseline absolute neutrophil count**^e^**	< 0.5 ×10^3^/µL	34 (27.2)
Baseline platelet count	<50 ×10^3^/µL	81 (64.8)
	<20 ×10^3^/µL	38 (30.4)
Glomerular filtration rate^f^	<60 mL/min/1.73 m^2^	21 (16.8)
	<30 mL/min/1.73 m^2^	7 (5.6)
HSV prophylaxis	Acyclovir 250 mg/m^2^ IV every 8 h	10 (8.0)
	Acyclovir 400 mg BID	44 (35.2)
	Acyclovir ≥800 mg BID	16 (12.8)
	Valacyclovir 500 mg BID	32 (25.6)
	Valacyclovir 500 mg QD	10 (8.0)
	Foscarnet^g^	6 (4.8)
	Others^h^	7 (5.6)

Abbreviations: ATG, antithymocyte globulin; BID, twice per day; HCT, hematopoietic cell transplant; HSV, herpes simplex virus; IV, intravenous; PTCy, posttransplant cyclophosphamide.

^a^Others: myelofibrosis (N = 3), aplastic anemia (N = 5), GATA-2 deficiency (N = 1), plasmacytoid dendritic cell neoplasm (N = 1).

^b^Three patients received ATG.

^c^Seventeen patients received ATG.

^d^ATG, PTCy (1 patient each).

^e^Nineteen patients with absolute neutrophil count < 0.5 ×10^3^/µL and platelets <20 ×10^3^/µL.

^f^Calculated using estimated glomerular filtration rate in 112 patients and the Cockcroft-Gault equation in 13.

^g^Foscarnet given for cytomegalovirus, secondary prophylaxis (5 patients), and induction (1 patient).

^h^Acyclovir 400 mg daily (N = 1), famciclovir 500 mg daily (N = 6).

### Virologic Characteristics

Table 2 summarizes the sites of HSV infection and virologic characteristics. Eighty-two percent of patients had orofacial infections, and 18% of patients had genital and/or perineal infections. Eight patients (6.4%) had cutaneous lesions in ≥2 noncontiguous sites. Fifteen patients had systemic involvement (respiratory tract, gastrointestinal tract, central nervous system, and cornea).

**Table 2.** Herpes Simplex Virus Infection Types, Sites of Lesions, and Antiviral Resistance

Characteristic		Total N = 125 N (%)
Diagnostic method	Polymerase chain reaction	105 (84.0)
	Culture	12 (9.6)
	Immunohistochemistry	8 (6.4)
Serotype	Herpes simplex virus type 1	89 (71.2)
	Herpes simplex virus type 2	21(16.8)
	Not available	15 (12.0)
Mucocutaneous^a^	Oral/facial	103 (82.4)
	Genital/perianal	23 (18.4)
	Other cutaneous^b^	5 (4.0)
System involvement	…	15 (12.0)
Respiratory	Pneumonitis, tracheitis, epiglottitis, laryngitis (1 each)	4 (3.2)
Gastrointestinal	Esophagitis	6 (4.8)
Central nervous system	…	1 (0.8)
Cornea	…	4 (3.2)
Number of antiviral resistance assays performed**^c^**	Acyclovir	104 (83.2)
	Phenotypic assays	98 (78.4)
	Genotypic assays	6 (4.8)
	Foscarnet	77 (61.6)
	Phenotypic assays	77 (61.6)
	Genotypic assays	0 (0.0)
Number of patients with laboratory confirmed resistance^d^	…	…
	Acyclovir resistance	85 (68.0)
	Foscarnet resistance	4 (3.2)

^a^Eight patients had ≥2 noncontiguous sites; oral/facial + genital/perianal (N = 3), oral/facial + cornea (N = 2), oral/facial + neck + cornea (N = 1), oral/facial + finger + cornea (N = 1), and oral/facial + neck (N = 1).

^b^Other: Left upper eyelid (N = 1), lower back (N = 1), neck (N = 2), finger (N = 1).

^c^108 patients were tested for antiviral resistance including 73 for acyclovir and foscarnet, 31 for acyclovir only, and 4 for foscarnet only.

^d^Based on cutoff inhibitory concentration (IC_50_) established by the testing laboratory.

Of 104 patients tested for acyclovir resistance, 85 (81.7%) had laboratory-confirmed acyclovir resistance, by phenotypic assay (79 patients) or genotypic assays (6 patients) (Table 2). The median IC_50_ for acyclovir was 5.6 µg/mL (Q1, Q3: 3.7, 14.1; minimum, maximum: 2.1, 64.0). Four patients had laboratory-confirmed foscarnet resistance, by phenotypic assays. In 3 of the 4 patients, foscarnet resistance testing was performed after a median of 25 days (minimum, maximum: 20, 37) of foscarnet therapy. The fourth patient had remote history of foscarnet exposure and foscarnet IC_50_ was 130.9 µg/mL (IC_50_ cutoff ≥100 µg/mL).

### Treatment patterns

#### Our First Objective was to Describe the Treatment Patterns for Refractory HSV

**Figure 1.**
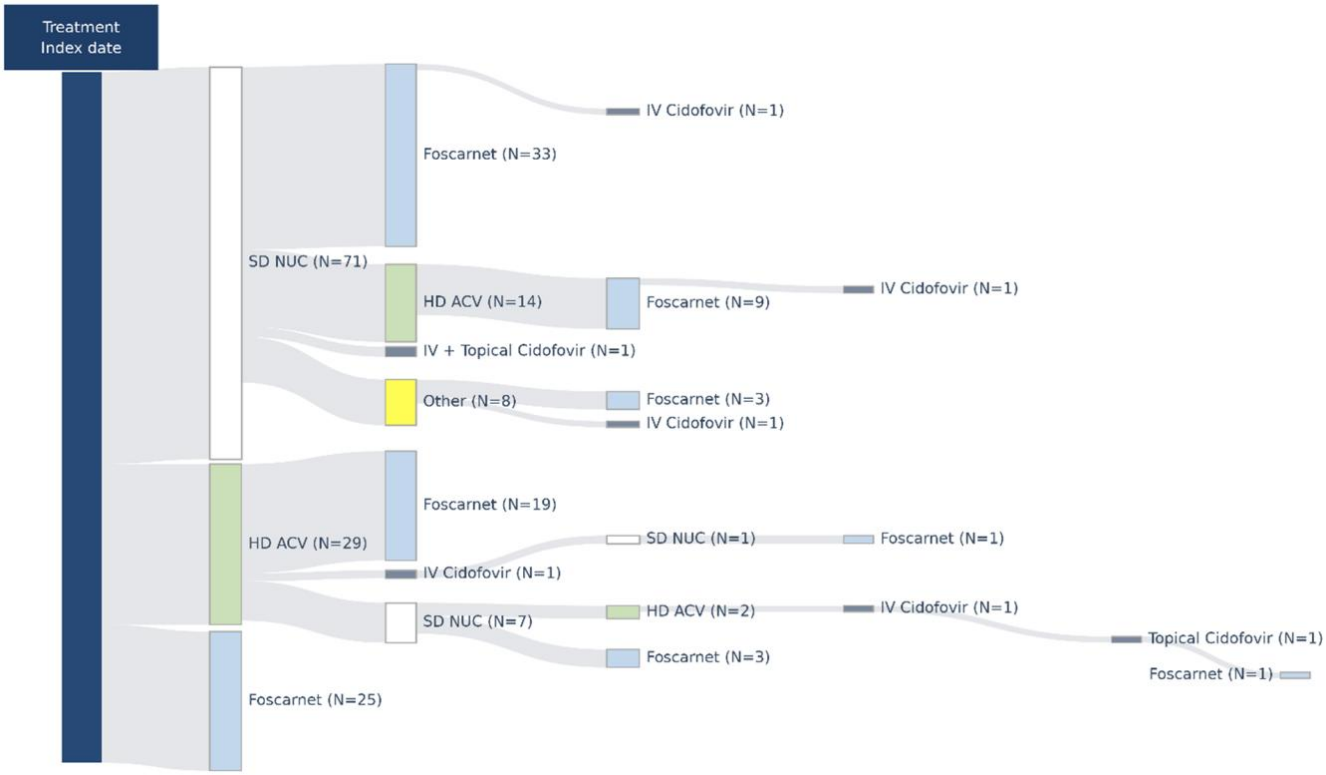
Treatment pathways up to first course foscarnet or intravenous cidofovir. Sequence of antivirals up to first course of foscarnet or IV cidofovir. The first anti-herpes simplex virus treatment was nucleoside analogue (NUC) in 100 patients (71 patients received standard doses NUC and 29 patients received high dose [HD] IV acyclovir as first therapy). Of 43 patients who received HD IV acyclovir, 33 patients (76.7%) eventually received foscarnet. SD NUC, standard dose nucleoside analogue; ACV, acyclovir. Vertical lines: dark blue: treatment index date; white: NUC: standard dose nucleoside analogue therapy; green: HD IV acyclovir; light blue: foscarnet; black: IV cidofovir. Antiviral courses following the first foscarnet and first IV cidofovir are not shown.

Anti-HSV treatment started a median of 1 day (Q1, Q3: 0, 2; minimum, maximum: 0, 14) from HSV diagnosis. Figure 1 shows the sequence of HSV antivirals from start of anti-HSV therapy until initiation of foscarnet and/or IV cidofovir. First anti-HSV treatment was a NUC (standard or high dose) in 100 patients (80.0%) and foscarnet in 25 patients (20.0%). Fourteen patients (11.2%) received only NUC because of patient preference and/or physician decision. All 14 patients met criteria for refractory HSV, and 8 of 14 (57.1%) had confirmed acyclovir resistance.

Seventy-seven percent of patients who received HD acyclovir were switched to second-line therapies. The most common second line therapy was foscarnet in 94 patients (75.0%). The median time from the treatment index dated to the start of the first course of foscarnet was 12 days (Q1, Q3: 0, 23). The patient disposition at the end of each sequential antiviral course is shown on Supplementary Figure 1.

### Primary Endpoint

Sixty-nine patients (55.2%) achieved our primary endpoint of complete healing. Forty patients (32.0%) died on therapy and 16 patients (12.8%) were censored before healing.

**Figure 2.**
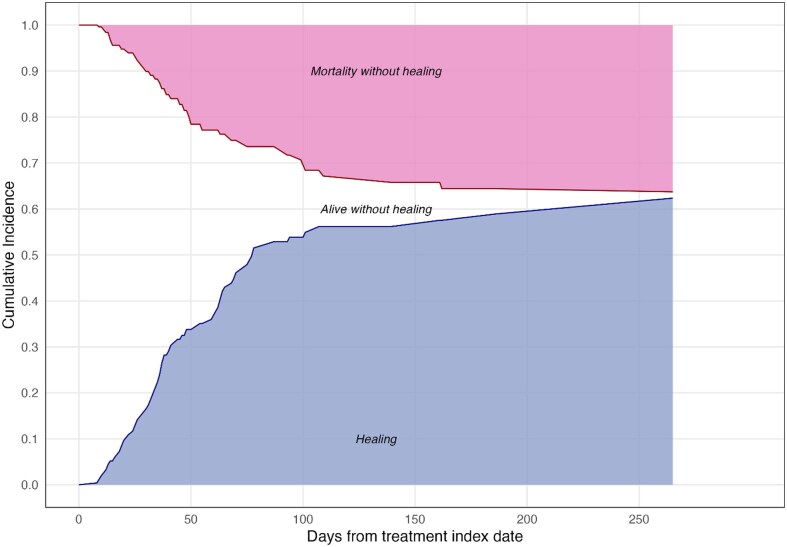
Time to complete lesion healing from start of anti-herpes simplex virus therapy (treatment index date). Of 125 patients, 69 patients (55.2%) healed at a median of 38 days (Q1, Q3: 26, 65; range 257) from treatment index date. In this plot, the lower curve (blue) represents the cumulative incidence of lesion healing from HSV treatment index date, with death as a competing risk. Patients who discontinued antiviral treatment prior to lesion healing were censored. The upper curve (pink) represents the cumulative incidence of overall mortality among patients without lesion healing. The middle portion represents the proportion of patients alive without lesion healing.

Complete healing was achieved at a median of 38 days (Q1, Q3: 26, 65; range: 257) from the treatment index date (Figure 2). The cumulative incidence of healing was 16.2% at day 28, 31.2% at day 42, and 53.4% at day 90 from the treatment index date.

Sensitivity analyses were done to evaluate the robustness of time-to-healing estimates across clinically relevant subgroups. Among patients excluding those treated with pritelivir (N = 121), the median time to healing was 38 days (Q1, Q3: 26, 65), with cumulative incidence of healing of 16.6% at day 28, 31.8% at day 42 and 53.9% at day 90.

In the contemporary cohort (2017–2024; N = 90), the median time to healing was 37.5 days (Q1, Q3: 23, 63), with cumulative incidence of healing of 19.1% at day 28, 30.6% at day 42 and 52.6% at day 90.

Among patients excluding the 14 patients who only received NUC therapy (N = 111), the median time to healing was 38 days (Q1, Q3: 26, 65), with cumulative incidence of 16.2% at day 28, 31.2% at day 42, and 53.4% at day 90.

Overall, estimates of time to healing were consistent across all sensitivity analyses.

The median follow-up time among censored patients was 76 days (Q1, Q3: 35.5, 97.5), exceeding the median time to healing in the overall cohort (38 days; Q1, Q3: 26, 65), indicating that censored observations contributed substantial follow-up time and were unlikely to bias the estimates of time to healing.

Supplementary Figure 2 shows the Kernel density plot of distribution of time to healing among the 69 patients who achieved clinical healing during follow-up. Time to healing was defined as the interval from treatment index date to date of complete healing. The most frequent observed healing time was approximately 30.8 days (dashed vertical line).

### Duration of Treatment of Second Line Therapies

Ninety-four patients received foscarnet, 6 received IV cidofovir, and 21 received topical antivirals for a total of 109 foscarnet courses, 6 IV cidofovir courses, and 28 topical antiviral courses. Supplementary Table 2 summarizes the exposure per course and antiviral type and total antiviral days. The treatment duration was a mean (SD) of 21.8 (13.8), 20.3 (10.1), and 41.0 (37.5) days for foscarnet, IV cidofovir, and topical antivirals, respectively. Courses of combination antivirals are summarized in Supplementary Table 3.

### Toxicities During Foscarnet or Cidofovir Treatment

Supplementary Table 4 summarizes toxicities during foscarnet or cidofovir treatment. Among 109 foscarnet courses, ≥1 toxicity was reported during 75 courses (68.8%). Discontinuation because of toxicity was reported in 33 (30.3%) courses. The most common toxicity or intolerance was renal function abnormalities reported during 44 courses (40.4%), followed by electrolyte abnormalities and gastrointestinal symptoms (nausea and anorexia).

Among 6 IV cidofovir courses, renal function abnormalities were reported during 3 courses (50%). One of 6 courses of IV cidofovir was discontinued because of the renal function abnormalities.

### HSV Recurrence

**Figure 3.**
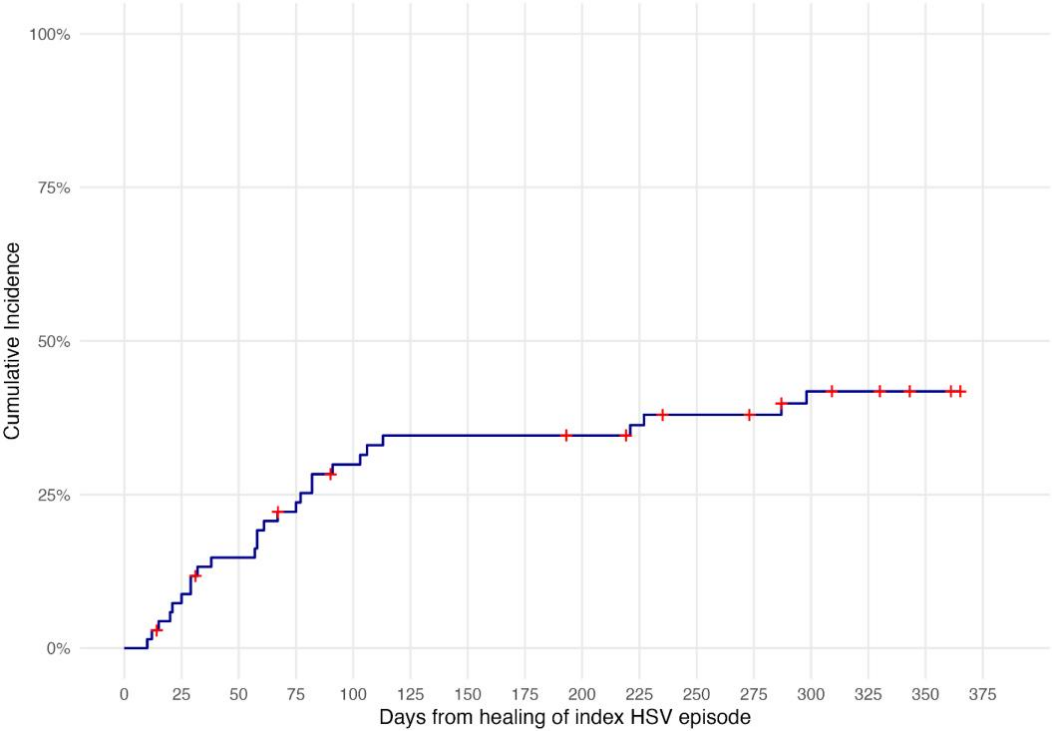
Time to first recurrence among the 69 patients who had complete healing of the index episode. Time to first HSV recurrence among the 69 patients who achieved complete healing of the index HSV episode. Twenty-seven patients (39.1%) had recurrent episodes with the first recurrent episode occurring at a median of 61 days (Q1, Q3: 29, 97; minimum, maximum: 10, 298). Day 0 is the day of complete healing of the index HSV episode. Patients were followed up to 1 year from first index HSV episode lesion healing. Red crosses indicate patients who were censored. Death was considered a competing risk for HSV recurrence.

Sixty-nine patients achieved complete healing of the index HSV episodes. These patients were followed for HSV recurrence for up to 1 year after healing, death, or last follow up. At 1 year, 22 patients (31.9%) died and 27 patients (39.1%) had 1 or more recurrent HSV episode(s). Figure 3 shows the time to first HSV recurrence. The time to first recurrence was a median of 61 days (Q1, Q3: 29, 97; minimum, maximum: 10, 298). Of a total of 30 recurrent episodes, 7 had acyclovir resistance testing and acyclovir resistance was confirmed in 5 episodes.

## DISCUSSION

This multicenter, retrospective, observational study describes real-world treatment patterns and clinical outcomes of refractory mucocutaneous HSV infections in a cohort of 125 HCT recipients from 7 major HCT centers in the United States. More than 75% of the patients presented with refractory orofacial lesions caused by HSV-1. All patients developed HSV infection during HSV prophylaxis. Of 104 patients tested for acyclovir resistance, 81.7% had laboratory-confirmed acyclovir resistance.

Acyclovir resistance results from mutations in the viral thymidine kinase gene, which prevent phosphorylation and confer resistance to acyclovir, and typically, these strains retain sensitivity to foscarnet and cidofovir. Mutations in the viral DNA polymerase gene confer resistance to foscarnet and are typically encountered after foscarnet exposure [25–27]. Resistance is more common in immunocompromised patients and typically arises after prolonged exposure to acyclovir [5]. During the study period, all but 1 participating center used phenotypic assays for acyclovir resistance, which take weeks to be performed and may not be readily accessible.

Treatment decisions for refractory mucocutaneous HSV are complex and highly individualized. Published guidelines encompass a wide range of hosts and are limited by the lack of controlled studies or approved therapies for refractory HSV [28]. Foscarnet requires daily IV administration; consequently, initiation of foscarnet may be deferred until the lesions worsen and/or hospitalization is deemed necessary based on physician judgment and patient preferences. Systemic cidofovir requires weekly IV administration and, although it could theoretically be given in the outpatient setting, concerns for nephrotoxicity and limited efficacy data hinder its applicability for refractory HSV.

We report real-world, contemporary, clinical practices for refractory HSV in HCT. Our definitions for refractory HSV are in alignment with the recently published definitions for clinical trials with respect to requirement of 7 days of therapy for definition of clinical failure [8] but are broader and include any appropriately dosed anti-HSV therapy, rather than strictly nucleoside analog therapy. The published definitions are developed specifically to facilitate inclusion criteria and endpoints for clinical trials. In contrast, our study was meant to capture the heterogeneity of real-world practices.

One-third of patients received high-dose IV acyclovir before second-line therapies, despite limited data for efficacy of high-dose IV acyclovir for acyclovir-resistant HSV. The number of antiviral courses and duration of exposure highlight the burden of therapy for refractory HSV infections.

Overall, 55% of patients achieved complete healing at a median of 38 days of anti-HSV therapy. Results were consistent across clinically relevant subgroups. Foscarnet was the most common second-line therapy, administered in a health care setting (hospital and/or hospital-associated infusion clinic) for a mean duration of 21.8 days. Renal function abnormalities and dose-limiting toxicities occurred frequently, consistent with prior reports [9, 29–31]. Health care resource utilization and costs associated with foscarnet were beyond the scope of this study but should be quantified in future studies especially in view of pritilevir that is orally bioavailable with a favorable safety profile [24].

Our cohort comprised highly complex and heavily immunosuppressed HCT patients with multiple risk factors for refractory HSV [14, 32–34]. Thus the prolonged time to healing may reflect not only persistent HSV replication but also impaired tissue repair associated with cytotoxic chemotherapy, mucositis, systemic corticosteroid exposure, and chronic graft-versus-host disease [35–37]. Thirty-nine percent of patients had recurrent HSV infections during the follow-up period, suggesting persistent immune deficits.

Our study has several limitations inherent to retrospective studies, and specifically to HSV infections. First, there was heterogeneity in anti-HSV treatments, and a prolonged study period (2009–2024) during which management practices may have evolved. To address these limitations, we performed sensitivity analyses restricted to clinically relevant subgroups. These analyses yielded consistent results, supporting our primary findings. Second, the quality of data and reporting of clinical outcomes was based on free text interpretation from standard of care assessments, with potential variability within and across institutions. Third, the adjudication of complete healing for HSV requires visual inspection. Subjectivity of the examiner, full visualization of all lesions and variation in frequency of clinic visits may have impacted the reporting of complete healing. Complete healing may be difficult to assess in the setting of concomitant mucositis or oral graft-versus-host disease. Quantitative clinical responses could not be captured because of a lack of standardized assessments or measurements. Fourth, reporting of toxicity could be prone to misclassification bias because no laboratory cutoff values were used. Last, our study was conducted in major academic HCT centers with complex patients and readily available diagnostic assays for HSV diagnosis and acyclovir resistance testing. Further studies are needed to validate our findings in different settings.

Acknowledging these limitations, our data demonstrate the real-world challenges with currently available therapies for refractory HSV and underscore the unmet need for effective and well-tolerated antivirals for refractory HSV infections in HCT recipients.

## Supplementary Material

ofag377_Supplementary_Data

## Supplementary Data

Supplementary materials are available at *Open Forum Infectious Diseases* online. Consisting of data provided by the authors to benefit the reader, the posted materials are not copyedited and are the sole responsibility of the authors, so questions or comments should be addressed to the corresponding author.
