# Supplementary material for: Refractory Mucocutaneous Infections by Herpes Simplex Virus (HSV) in Hematopoietic Cell Transplant Recipients: A Real-world, Multicenter Study
Source: Open Forum Infect Dis. 2026 Jun 19;13(7):ofag377. doi: 10.1093/ofid/ofag377 (PMC13386174; doi:10.1093/ofid/ofag377)
Supplement: ofag377_Supplementary_Data [file ofag377_supplementary_data.docx]

**Supplemental Tables**

**Table S1.** Anti-herpes simplex virus therapies

|  | **Antiviral^1^** | **Route of administration** |
| --- | --- | --- |
| **Standard dose nucleoside analogue** |  |  |
|  | Acyclovir ≤15mg/kg/day | Intravenous |
|  | Ganciclovir | Intravenous |
|  | Acyclovir | Oral |
|  | Famciclovir | Oral |
|  | Valacyclovir | Oral |
|  | Valganciclovir | Oral |
|  |  |  |
| **High dose nucleoside analogue** |  |  |
|  | Acyclovir ≥30mg/kg/day | Intravenous |
|  |  |  |
| **Second line** |  |  |
|  | Foscarnet | Intravenous |
|  | Cidofovir | Intravenous |
|  | Cidofovir | Topical |
|  | Imiquimod | Topical |

^1^Doses appropriate for HSV treatment^,^ adjusted for creatinine clearance.

**Table S2. Duration of treatment with second line therapies**

| Exposure (days) | **Foscarnet**  (109 courses) | **IV Cidofovir**  (6 courses) | **Topical antivirals^1^**  (28 courses) |
| --- | --- | --- | --- |
|  |  |  |  |
| Mean (Standard deviation) | 21.8 (±13.8) | 20.3 (±10.1) | 41.0 (±37.5) |
| Median | 20.0 | 19.0 | 36.5 |
| Q1, Q3 | 11.0, 29.0 | 15.8, 23.8 | 16.3, 51.0 |
| Total antiviral days | 2,376 | 122 | 1,147 |

Footnote: ^1^ Includes topical cidofovir and imiquimod. Twenty of the 28 courses included combination of topical and systemic antivirals (**Table S3**). Ninety-four patients received foscarnet, 6 patients received intravenous cidofovir and 21 patients received topical antivirals.

Abbreviation: Q: quartile

**Table S3.** Antiviral courses with >1 antiviral (total n=23)

| **Combination** | **Number of patients** | **Number of courses** | **Total antiviral days** | **Mean number of antiviral days** |
| --- | --- | --- | --- | --- |
| **Systemic antivirals** |  |  |  |  |
| Foscarnet + HD IV acyclovir | 1 | 1 | 7 days |  |
| Foscarnet + Valacyclovir | 3 | 3 | 46 days | 15.3 days |
|  |  |  |  |  |
| **Topical cidofovir +** |  |  |  |  |
| Topical cidofovir +Valacyclovir | 8 | 8 | 261 days | 32.6 days |
| Topical cidofovir + SD IV acyclovir | 1 | 1 | 17 days |  |
| Topical cidofovir + Foscarnet | 5 | 5 | 107 days | 21.4 days |
| Topical cidofovir+ IV cidofovir | 1 | 1 | 37 days |  |
|  |  |  |  |  |
| **Topical imiquimod+** |  |  |  |  |
| Topical imiquimod +Foscarnet | 1 | 1 | 5 days |  |
| Topical imiquimod +Topical cidofovir +Valacyclovir+ Foscarnet | 1 | 1 | 38 days |  |
| Topical imiquimod +Valacyclovir | 1 | 1 | 58 days |  |
| Topical imiquimod + Topical cidofovir | 1 | 1 | 19 days |  |

Abbreviations: HD: high dose, SD: Standard dose, IV: intravenous.

Footnote: SD IV acyclovir ≤15mg/kg/day; HD IV acyclovir ≥30mg/kg/day both adjusted for creatinine clearance.

**Table S4.** Toxicities reported during foscarnet or intravenous cidofovir treatment.

|  | **Foscarnet**  **109 courses**  **N (%)** | **IV Cidofovir**  **6 courses**  **N (%)** |
| --- | --- | --- |
| Any toxicity | 75 (68.8) | 3 (50.0) |
| Toxicity leading to discontinuation | 33 (30.3) | 1 (16.7) |
| Renal function abnormalities | 44 (40.4) | 3 (50.0) |
| Electrolyte abnormalities | 14 (12.8) |  |
| Gastrointestinal | 7 (6.4) |  |
| Other^#^ | 10 (9.2) |  |

Footnote^: #^reported in <5%: volume overload (N=4), confusion (N=1), myelosuppression (N=2), penile ulceration (N=1), and logistic challenges (N=2).

**Supplemental Figures**

**Figure S1.** Patient disposition at each sequential antiviral course (AV) starting from treatment index date.


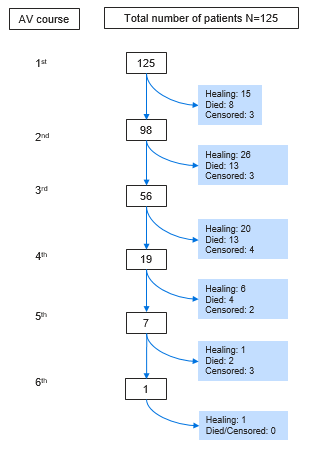


Clinical outcomes at the end of each antiviral course starting from first anti-HSV therapy. Of 69 patients that achieved complete healing, 52 patients (75.3%) healed between the 2^nd^ and 4^th^ antiviral course.

Abbreviation: AV: antiviral

**Figure S2.** Density distribution of time to healing among patients who achieved complete healing (N = 69)


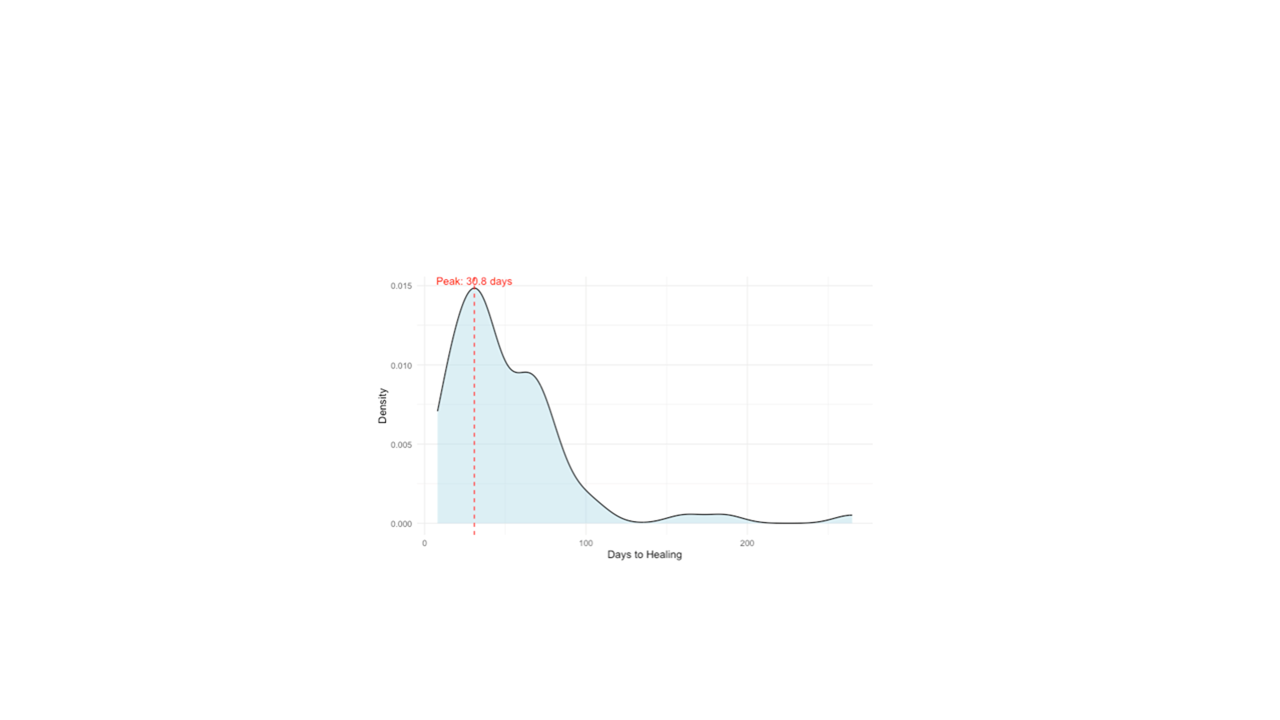


Legend: Kernel density plot showing the distribution of time to healing among the 69 patients who achieved complete healing during follow-up. The dashed vertical line indicates the peak density (approximately 30.8 days), representing the most frequent observed healing time.
